# Preparation of Bis[(3-ethyl-3-methoxyoxetane)propyl]diphenylsilane and Investigation of Its Cationic UV-Curing Material Properties

**DOI:** 10.3390/polym13152573

**Published:** 2021-08-02

**Authors:** Yuansheng Liu, Biwu Huang, Wenbin Zhou, Weiqing Chen, Yang Wu

**Affiliations:** School of Materials Science and Engineering, Nanchang University, Nanchang 330031, China; lys1579777@sina.com (Y.L.); z15270877174@163.com (W.Z.); Wqchen@ncu.edu.cn (W.C.); wuyang@ncu.edu.cn (Y.W.)

**Keywords:** UV curing, photosensitive resin, hydrosilylation reaction

## Abstract

Precusor EHO(3-ethyl-3-hydroxymethyloxetane) was synthesized with diethyl carbonate and trihydroxypropane as the main raw materials. Intermediate AllyEHO(3-ethyl-3-allylmethoxyoxetane) was synthesized with 3-ethyl-3-hydroxymethyloxetane and allyl bromide as the main raw materials. Prepolymer bis[(3-ethyl-3-methoxyoxetane)propyl]diphenylsilane was synthesized with 3-ethyl-3-methoxyoxetane)propyl and diphenylsilane. Photoinitiator triarylsulfonium hexafluoroantimonate of 3% was added to the prepolymer, and a novel kind of the photosensitive resin was prepared. They were analyzed and characterized with FTIR and ^1^H-NMR. Photo-DSC examination revealed that the bis[(3-ethyl-3-methoxyoxetane)propyl]diphenylsilane has great photosensitivity. The thermal properties and mechanical properties of the photosensitive resin were examined by TGA and a microcomputer-controlled universal material testing machine, with thermal stabilities of up to 446 °C. The tensile strength was 75.5 MPa and the bending strength was 49.5 MPa. The light transmittance remained above 98%.

## 1. Introduction

Ultraviolet (UV)-initiated photopolymerization systems have been fast-growing and widely used in many applications where the traditional thermal curing system has been almost impossible for decades, because the polymerizations can be carried out solvent-free at room temperature [1,2,3,4,5]. UV curing technology has the advantages of fast curing speed, less pollution and energy efficiency and ambient temperature requirement [6,7,8]. UV-curable technology is applied extensively in the printing industry, biomedical applications, adhesives, 3D printing and the information industry [9,10,11,12].

UV curing technology mainly has two curing mechanisms, free radical curing [13,14] and cationic curing [15,16,17]. The free radical curing system has developed a variety of prepolymer monomers, which has the advantages of fast reaction speed and easy adjustment of the cured film performance. However, the shortcomings such as large volume shrinkage and poor adhesion restrict its development [18,19,20,21]. The advantages of cationic photopolymerization are wear resistance, high hardness, small volume shrinkage, strong adhesion [22,23,24]. The biggest advantage of cationic photopolymerization is that it does not exhibit oxygen inhibition like radical-based systems do [25,26,27,28].

With the rapid development of UV-curing technology, cationic UV-curing systems are attracting increased attention because of their outstanding advantages. Cycloaliphatic epoxides, oxetanes and bisphenol A type epoxy compound are used as prepolymers in the cationic curing systems [29,30]. Compared to the cycloaliphatic epoxides of cationic UV-curing systems, oxetane polymerization have a lower viscosity, lower toxicity, higher curing rate and higher thermal stability [30,31]. However, the photosensitivity of oxetanes is worse than the cycloaliphatic epoxides, which limits its scope of application for oxetanes [32,33,34,35].

Since Rhone Poulenc synthesized UV-curable silicone-modified acrylate in 1979, more and more people have begun to study the subject of combining UV-curable resins with silicone materials [36]. Scholars have introduced silicone materials into UV-curable resins, and can have advantages such as high weather resistance, low surface tension, low viscosity and low Tg of silicone materials to improve the weather resistance [37], thermal stability [38] and hardness [39] of UV-curable materials, also giving the coating better stain resistance [40]. However, such a modification has been accomplished by the addition of silicone benzene materials with oxetanes systems that, to the best of our knowledge, is presented for the first time in literature. It can increase the competitiveness of UV-curable materials in the field of 3D printing.

In this paper, diethyl carbonate and trihydroxypropane were used to synthesize the precursor EHO(3-ethyl-3-hydroxymethyloxetane); the precursor and allyl bromide as raw materials were used to generate the intermediate AllylEHO(3-ethyl-3-allylmethoxyoxetane); and finally prepolymer bis[(3-ethyl-3-methoxyoxetane)propyl]diphenylsilane was synthesized by AllylEHO and diphenylsilane. A series of photosensitive resin products were prepared with photoinitiator triarylsulfonium hexafluoroantimonate of 3% and prepolymer, which were subjected to Photo-DSC test, TG test, mechanical performance test and light transmittance.

## 2. Experimental

### 2.1. Materials

Diethyl carbonate (99.7%), trihydroxypropane (99.7%), anhydrous magnesium sulfate were purchased from Sinopharm Chemical Reagent Co., Ltd., Shanghai, China. Potassium hydroxide, allyl bromide (99.7%) were purchased from Tianjin Damao Chemical Reagent Factory, Tianjin, China. Dichloromethane (99.7%) and tetrabutylammonium bromide (99.7%) were purchased from Xilong Chemical Co., Ltd., Guangdong, China. Toluene (99.9%) was purchased from Nanjing Runsheng Petrochemical Co., Ltd., Nanjing, China. Cationic photoinitiator triarylsulfonium hexafluoroantimonate (99.7%) was purchased from Dow Union Carbide, namely, diphenylsilane (99.7%) was purchased from Beijing Bailingwei Technology Co., Ltd., Beijing, China. Tris(triphenylphosphine) rhodium chloride (99.7%) was purchased from Shanghai Yishi Chemical Co., Ltd., Beijing, China. All of them are analytically pure. E-51 (98%) was purchased from Shanghai Resin Factory, 3,4-Epoxycyclohexylmethyl-3′,4′- epoxy cyclohexane carboxylate (2021P) (98%) was purchased from Daicel (China) Investment Co., Ltd., Shanghai, China.

### 2.2. Characterization Methods

^1^H-NMR spectra of synthesized oxetanes have been recorded on a 400MR DD2 Nuclear Magnetic Resonance Spectrometer (American Agilent Technologies Co., Ltd., Palo Alto, CA, USA), operating at 600 MHz using CDCl_3_ as a solvent and Tetramethylsilane (TMS) as an internal reference. The FTIR spectra have been recorded on Nicolet 5700 Smart Fourier Transformation Red External spectrometer (American Thermoelectric Nicolas Corporation., MA, USA), in a transmittance mode, in the range of 4000–400 cm^−1^ at a resolution of 4 cm^−1^. Photo-DSC measurements have been taken with a TA Instruments Q2000 Photo-DSC apparatus (TA Instruments, New Castle, PA, USA) equipped with a mercury lamp and UV-intensity of 35 mW/cm2, under the nitrogen atmosphere (flow rate was 50 mL/min), using aluminum sample pans. Thermogravimetric analysis (TGA) has been performed with TGA 4000 Thermogravimetric Analyzer (American PE Company., Waltham, MA, USA), in a range of 25 to 800 °C at a heating rate of 10 °C/min in a stream of nitrogen (60 mL/min). Mechanical properties have been taken with CMT4204 microcomputer-controlled universal material testing machine (Shenzhen New Sansi Company, Shenzhen, China) at room temperature. Light transmittance has been performed with CARY 100 UV-Vis Spectrophotometer (Agilent Technologies Co., Ltd., California, CA, USA) in ranges of 240–800 nm (chloroform).

### 2.3. Synthesis

#### 2.3.1. Synthesis of 3-Ethyl-3-hydroxymethyloxetane Monomer

**Figure 1:**
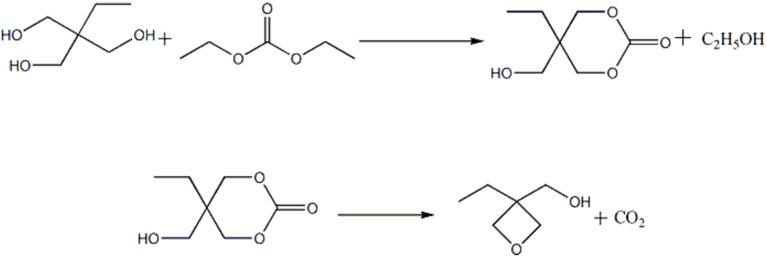
The synthetic route of 3-ethyl-3-hydroxymethyloxetane.

The weighed diethyl carbonate and trimethylolpropane were added to a 250 mL three-necked flask equipped with a thermometer, a stir bar, and a reflux condenser, and then an appropriate amount of methanol solvent and potassium hydroxide catalyst was added to the three-necked flask. Reflux reaction maintained for 1h at the temperature of 115 °C until the transesterification reaction of diethyl carbonate and trihydroxypropane had been completed and the intermediate of six-membered ring lactone structure was obtained. The reflux device was then converted into a distillation device, and the products produced by the reaction were collected by distillation. Then the reaction system was heated to 205–210 °C to remove CO_2_, vacuum distillation collected 130~135 °C/21.3 kPa fraction, and the collected fraction was the desired target product 3-ethyl-3-hydroxymethyloxetane. The synthesis path is shown in Figure 1.

#### 2.3.2. Synthesis of 3-Ethyl-3-allylmethoxyoxetane

**Figure 2:**
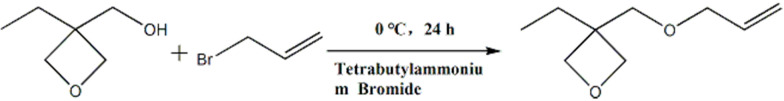
The synthetic route of 3-ethyl-3-allylmethoxyoxetane.

The distilled fraction 3-ethyl-3-hydroxymethyloxetane and 50% KOH aqueous solution were added to a three-necked flask equipped with a magnetic stirrer, a thermometer, a separatory funnel and a reflux tube. Tetrabutylammonium bromide was added to the mixture with vigorously stirring and allyl bromide in separatory funnel were added to the three-necked flask at a rate of one drop per second and stirred at 0 °C for 24 h. An appropriate amount of dichloromethane solution and distilled water were added to the reaction product and a separatory funnel was used to separate the phases. Organic phase was washed twice with distilled water, dried and filtered through anhydrous magnesium sulfate, and excess allyl bromide and extractant dichloromethane was removed by using a rotary evaporator. The residue was distilled under reduced pressure to collect 55–60 °C /0.4 kPa fraction, the product obtained is 3-ethyl-3-allylmethoxyoxetane (AllylEHO). The synthesis path is shown in Figure 2.

#### 2.3.3. Synthesis of Bis[(3-ethyl-3-methoxyoxetane)propyl]diphenylsilane

**Figure 3:**
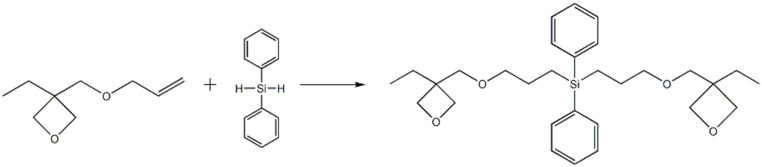
The synthetic route of bis[(3-ethyl-3-methoxyoxetane)propyl]diphenylsilane.

An appropriate amount of toluene,3-ethyl-3-allylmethoxyoxetane and tris(triphenylphosphine)rhodium chloride catalyst were accurately weighed into a 500 mL three-necked round bottom flask equipped with thermometer and condenser and a dropping funnel. The mixture of toluene and diphenylsilane in the dropping funnel was added into the flask while the temperature of mixture solution was kept at 88–92 °C, maintaining a titration rate of about 1.5 s for one drop. After the titration, the temperature was kept at 88–92 °C for 4–5 h until the reaction was completed. It would undergo a hydrosilylation reaction to obtain bis[(3-ethyl-3-methoxyoxetane)propyl]diphenylsilane. Then, under a vacuum condition, the mixture was heated to 100 °C, the solvent was distilled with a rotary evaporator to obtain the reacted product. The synthesis path is shown in Figure 3.

### 2.4. The Preparation of UV-Cured Spline

The prepolymer bis[(3-ethyl-3-methoxyoxetane)propyl]diphenylsilane and photoinitiator triarylsulfonium hexafluoroantimonate of 3% were mixed well, standing for a period of time until there were no bubbles in the mixed solution. The mixed solution was poured into the spline mold and evenly smeared the mixed solution on the glass sheets, putted them into the intelligent control light curing machine, and the light intensity was adjusted to 90% and cured for 60 s to obtain the UV-cured splines and films.

## 3. Results and Discussion

### 3.1. Structural Characterization

#### 3.1.1. FTIR

**Figure 4:**
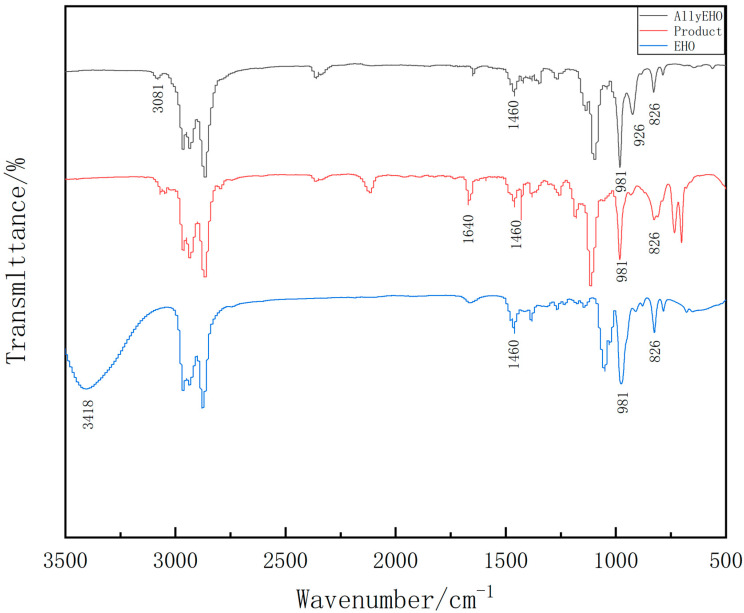
Infrared spectra of EHO, AllylEHO and bis[(3-ethyl-3-methoxyoxetane)propyl]diphenylsilane.

The FTIR spectrum of the EHO, AllylEHO and bis[(3-ethyl-3-methoxyoxetane)propyl]diphenylsilane, respectively, were displayed in Figure 4. In the curve of the EHO, peaks at 981 cm^−1^ characteristic of the anti-symmetric deformation vibration absorption peak of the four−membered ring in oxetane, 826 cm^−1^ characteristic of the symmetric deformation vibration absorption peak of oxetane, which shows there are four-membered ring in the molecular structure of the precursor and EHO is successfully synthesized. In the curve of the AllylEHO, new peaks are observed at 3081 cm^−1^, 1646 cm^−1^ and 926 cm^−1^ representative of the C-H aliphatic stretch of the characteristic absorption peaks of allyl groups compared with precursor EHO. Besides, peaks at 3411 cm^−1^ characteristic of hydroxyl stretching peak of EHO do not appear in the curve of AllylEHO, which illustrates hydroxyl reacting with bromine atom. Therefore, it can be concluded that the substance is 3-ethyl-3-allylmethoxyoxetane. In the FTIR spectrum for the bis[(3-ethyl-3-methoxyoxetane)propyl]diphenylsilane, a new peak is observed at 1640 cm^−1^ representative of the Si-C of the characteristic absorption peaks. Therefore, it can be concluded that the substance is bis[(3-ethyl-3-methoxyoxetane)propyl]diphenylsilane.

#### 3.1.2. ^1^H−NMR

**Figure 5:**
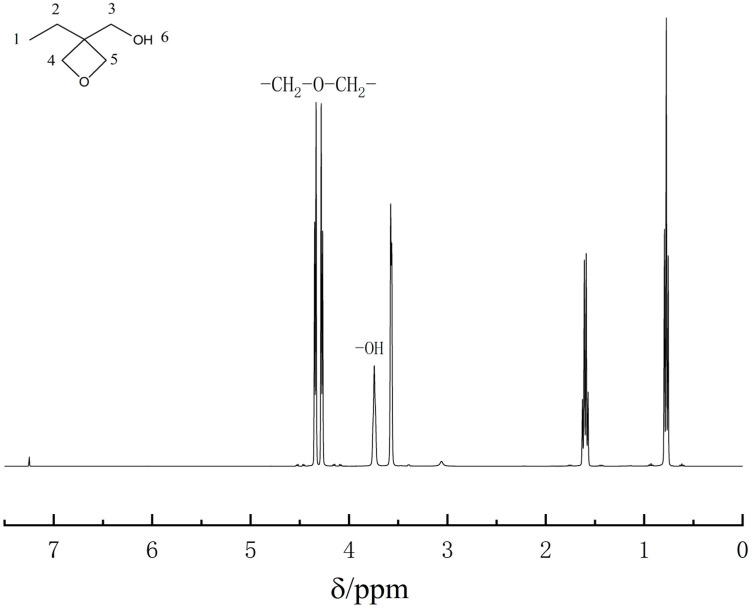
The hydrogen spectrum of 3-ethyl-3-hydroxymethyloxetane.

Bruker Advance 600 MHz nuclear magnetic resonance technology ^1^H-NMR also proves the successful synthesis of EHO(3-ethyl-3-hydroxymethyloxetane), as shown in Figure 5 and Table 1. Regarding the hydrogen of OH, the peak appeared at 3.74 ppm. The proton on oxygen heterocyclic which is formed of condensation of diethyl carbonate and trihydroxypropane have peaks at 4.27 and 4.33 ppm. The absorption peak area ratio of the six peaks is 3:2:2:2:2:1. They prove that the precursor EHO has been synthesized.

**Figure 6:**
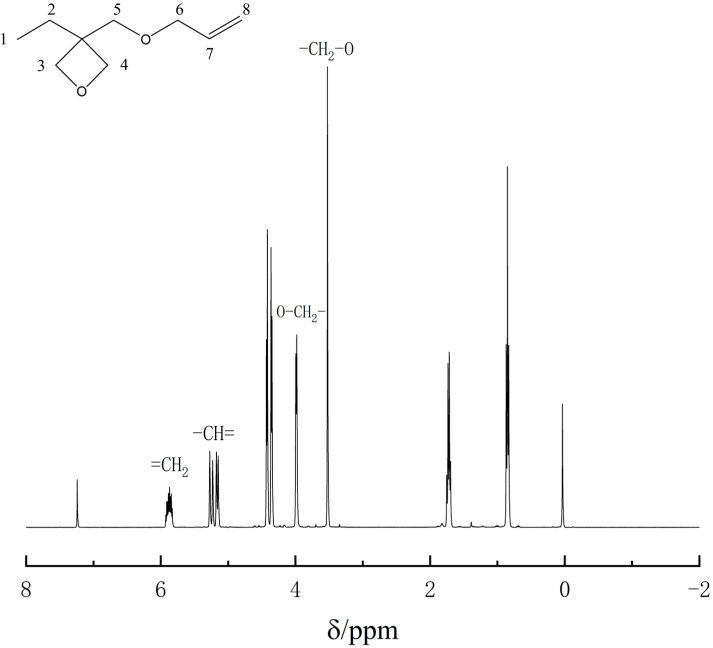
The hydrogen spectrum of 3-ethyl-3-allylmethoxyoxetane.

Bruker Advance 600 MHz nuclear magnetic resonance technology ^1^H-NMR also proves the successful synthesis of AllyEHO(3-ethyl-3-allylmethoxyoxetane), as shown in Figure 6 and Table 2. In ^1^H-NMR spectrum of AllylEHO, there is no characteristic peak with a chemical shift 3.74 ppm, but there are two chemical shifts of 3.49 and 3.92 ppm of ether bond, which present the substitution reaction allyl bromide and EHO. The chemical shifts of the propenyl double bond in the alkane are 5.25 ppm and 5.88 ppm, and the ratio of the eight peak absorption peak areas is 3:2:2:2:2:2:1:2. They prove that intermediate 3-ethyl-3-allyloxetane has been synthesized.

**Figure 7:**
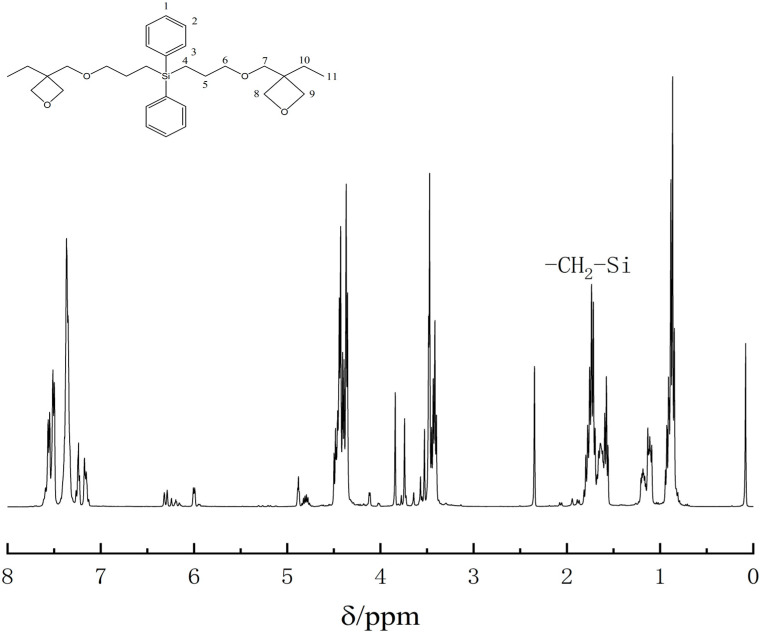
The hydrogen spectrum of bis[(3-ethyl-3-methoxyoxetane)propyl]diphenylsilane.

Bruker Advance 600 MHz nuclear magnetic resonance technology ^1^H-NMR also proves the successful synthesis of bis[(3-ethyl-3-methoxyoxetane)propyl]diphenylsilane, as shown in Figure 7 and Table 3. In ^1^H-NMR spectrum of bis[(3-ethyl-3-methoxyoxetane)propyl]diphenylsilane, there is no characteristic peak with a chemical shift of 4.7 ppm in the proton NMR spectrum of the product, indicating that there is no silicon-hydrogen bond in the product. There is no characteristic peak at the chemical shift 5.25 ppm and 5.88 ppm, indicating that there is no double bond in the product. At the same time, a characteristic peak with a chemical shift of 1.54 ppm has appeared, indicating the presence of silicon carbon in the product. The peak area ratio of the 11 absorption peaks is 3:2:2:2:2:2:2:2:2:2:1, which indirectly verify that the final product obtained from the experiment is bis[(3-ethyl-3-methoxyoxetane)propyl]diphenylsilane.

### 3.2. Photosensitivity of UV−Curable Materials

Photosensitivity is the most important performance index of UV−curable materials. Only when photo−curable materials have a fast curing rate can they have the practical value [41]. In this research, the Photo−DSC test was used to study the photosensitivity of synthetic resin.

**Figure 8:**
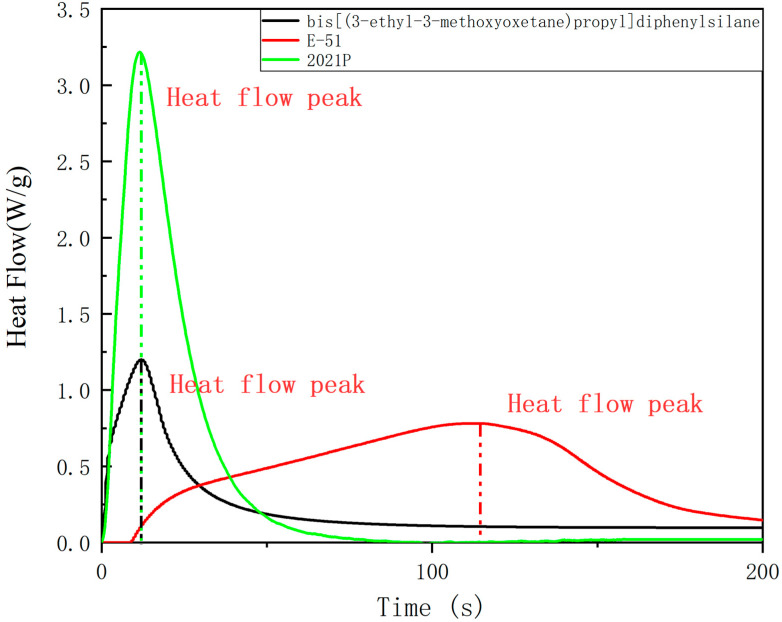
Heat flow of bis[(3-ethyl-3-methoxyoxetane)propyl]diphenylsilane,. E-51 and 2021 P.

**Figure 9:**
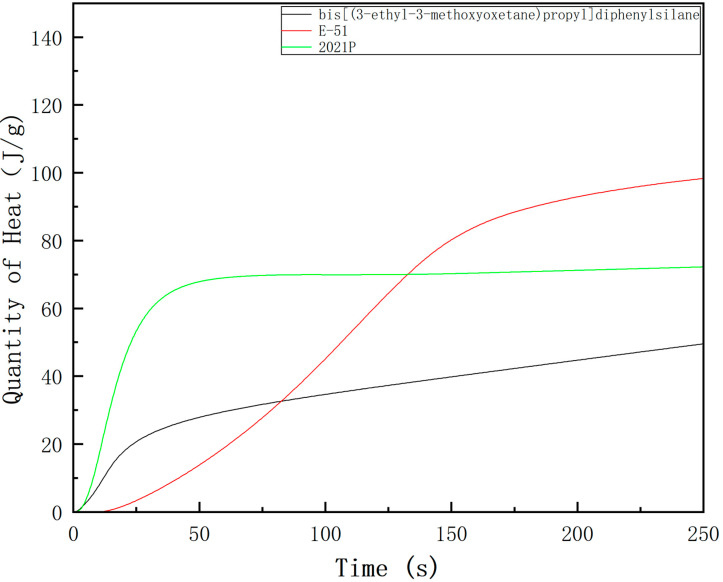
Heat quantity of bis[(3-ethyl-3-methoxyoxetane)propyl]diphenylsilane, E-51 and 2021 P.

In photo-DSC, the heat flow and total heat evolved are measured. Total heat evolved can be related to the final degree of cure and is measured as the area under the curve [42]. The heat flow and quantity of heat are shown in Figure 8 and Figure 9. To determine the photosensitivity of cycloaliphatic epoxides, oxetanes and bisphenol A type epoxy compound, we measured Photo−DSC traces of bis[(3-ethyl-3-methoxyoxetane)propyl]diphenylsilane, E-51 and 2021P (Figure 8) and get the derived date of them(Table 4). It reveals that the trace of the three showed one exothermic peak. The step of 2021P occurs in the time range of 0~52.8 s and the maximum reaction rate (t_max_) at 11.7 s; the step of bis[(3-ethyl-3-methoxyoxetane)propyl]diphenylsilane occurs in the time range of 0~80 s and the maximum reaction rate (t_max_) at 12.0 s; the step of E−51 occurs in the time range of 76~147.6 s and the maximum reaction rate (t_max_) at 113.4 s, indicating that the sample has great photosensitivity. Induction time and H_max_ can reflect the reactivity of a reactant to a certain extent. The induction time of bis[(3-ethyl-3-methoxyoxetane)propyl]diphenylsilane is 0.2 s, which is close to the 2021P(0.15 s) and far short of E-51(9 s). The H_max_ of bis[(3-ethyl-3-methoxyoxetane)propyl]diphenylsilane is 26.40 J/g, nearly half of 2021P(43.5 J/g) and one third of E−51(65.9 J/g). It can characterize bis[(3-ethyl-3-methoxyoxetane)propyl]diphenylsilane as having great reactivity. They show that the photosensitivity of bis[(3-ethyl-3-methoxyoxetane)propyl]diphenylsilane is close to 2021P, the great product 2021P in market, fully more than E-51.

### 3.3. Thermal Characterizations

**Figure 10:**
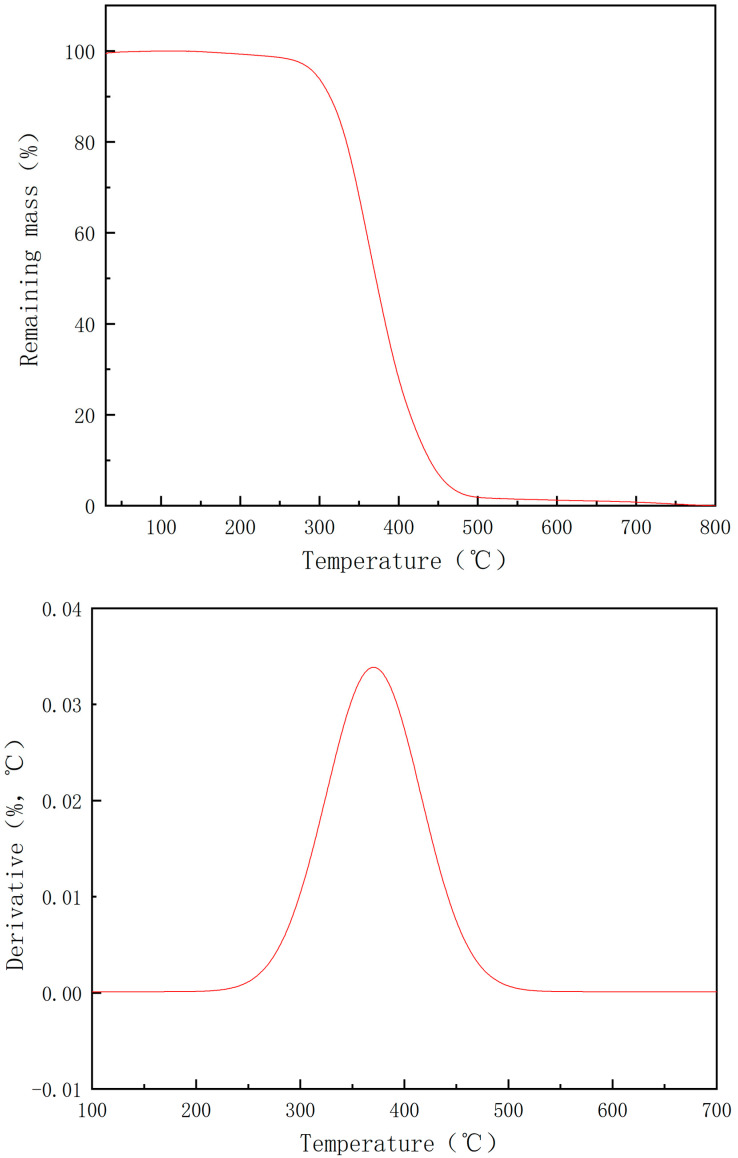
TG and DTG curves of the compound bis[(3-ethyl-3-methoxyoxetane)propyl]diphenylsilane.

The thermal stabilities of the prepared compounds were evaluated by thermogravimetric analysis (TG). The TG thermogram of the final product is displayed in Figure 10, as a representative example [43]. As can be seen from Figure 10, the decomposition takes place through two degradation steps depending on the molecular structure of the compound. The first step occurs in the temperature range of 230–330 °C and starts at 255 °C with maximum degradation rate (T_max_) at 370 °C indicating that the sample has a high thermal stability, while the second decomposition step occurs between 400 °C and 520 °C with maximum degradation rate of 446 °C. The results revealed that the investigated materials possess high thermal stabilities of up to 446 °C.

### 3.4. Testing of Mechanical Properties of UV−Curable Material

Tensile splines and bending splines prepared with prepolymer bis[(3-ethyl-3-methoxyoxetane)propyl]diphenylsilane and photoinitiator triarylsulfonium hexafluoroantimonate meet the standards of GB/T 1040-92 “Test Methods for Plastic Tensile Properties” and the standards of GB/T 9341–2008 “Determination of Plastic Bending Properties”. As shown in Table 5 and Table 6, the average value of tensile strength and bending strength were 75.5 MPa and 49.5 MPa at room temperature. There is silicon benzene in the molecular structure, and the molecular chain can be rearranged under the action of tension. They give the photosensitive resin good strength. The average value of elastic modulus was 19441.6 MPa and 1854.1 MPa at room temperature. The introduction of more flexible ether bonds in the molecular structure reduces the proportion of rigid benzene rings in the cured product, making the cured product softer and providing a better elastic modulus. All in all, the photosensitive resin prepared in the experiment has good mechanical properties.

### 3.5. Light Transmittance of UV−Curable Materials

**Figure 11:**
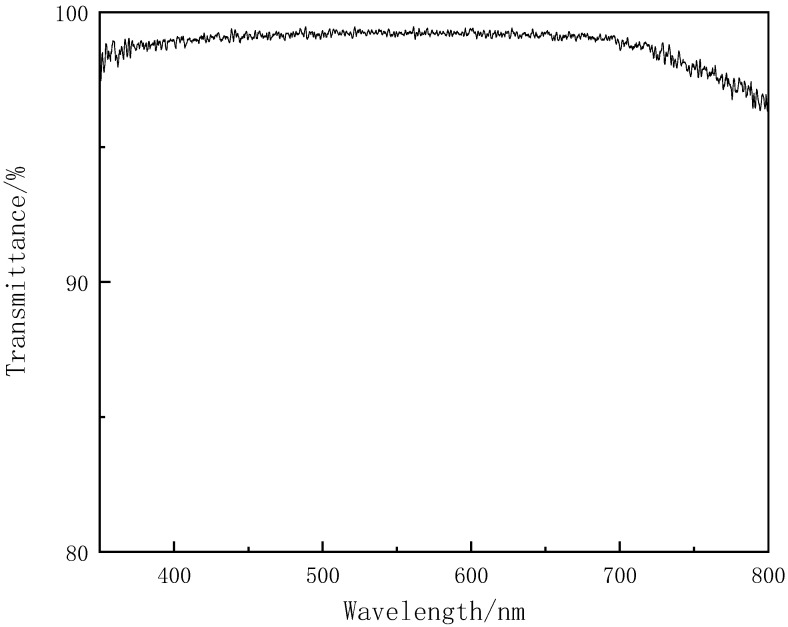
Light transmittance curves of the compound bis[(3-ethyl-3-methoxyoxetane)propyl]diphenylsilane.

Light transmittance of the prepared compounds evaluated by UV−visible spectrophotometer. As can be seen from Figure 11 and Table 7, in the wavelength range of 350 nm–700 nm, the light transmittance maintains above 98%. The cured film of bis[(3-ethyl-3-methoxyoxetane)propyl]diphenylsilane has good light transmittance in the range of wavelength of common UV curing light sources.

## 4. Conclusions

3-ethyl-3-hydroxymethyloxetane was synthesized by diethyl carbonate and trihydroxypropane in an alkaline environment; 3-ethyl-3-allylmethoxyoxetane was synthesized by allyl bromide and 3-ethyl-3-hydroxymethyloxetane; bis[(3-ethyl-3-methoxyoxetane)propyl]diphenylsilane was synthesized by 3-ethyl-3-allylmethoxyoxetane and diphenylsilane. Triarylsulfonium hexafluoroantimonate of 3% was added to bis[(3-ethyl-3-methoxyoxetane)propyl]diphenylsilane to prepare a new type of cationic photosensitive resin. Such a modification has been accomplished by the polymerization of oxetane with silicon benzene compound, which is presented for the first time in the literature, to the best of our knowledge.

The realization of such an approach has been confirmed using ^1^H-NMR, and FTIR spectroscopies. The photosensitivity has been determined by using photo-DSC, the introduction of diphenylsilane has improved the photosensitivity of oxtane. Besides, UV-cured product of bis[(3-ethyl-3-methoxyoxetane)propyl]diphenylsilane has great mechanical properties and thermal properties determined TG analyses and mechanical tests, because of the introduction of phenyl and silicon atoms. The light transmittance remains above 98%. Excellent photosensitivity of bis[(3-ethyl-3-methoxyoxetane)propyl]diphenylsilane meets the needs of 3D printing for resin materials, which further broadens the preparation range of UV-curable products, and has certain promotion and application value.

## Figures and Tables

**Table 1:** Analytical table of the ^1^H-NMR spectrum for the 3-ethyl-3-hydroxymethyloxetane.

Location of H	Chemical Shift/(δ/ppm)	Structural Formula
1	0.78	3H, –CH_3_
2	1.60	2H, –CH_2_–
3	3.57	2H, –CH_2_–
4	4.27	2H, –CH_2_–
5	4.33	2H, –CH_2_–
6	3.74	1H, –OH

**Table 2:** Analytical table of the ^1^H-NMR spectrum for the 3-ethyl-3-allylmethoxyoxetane.

Location of H	Chemical Shift/(δ/ppm)	Structural Formula
1	0.85	3H, −CH_3_
2	1.78	2H, −CH_2_−
3	4.36	2H, −CH_2_−
4	4.42	2H, −CH_2_−
5	3.49	2H, −CH_2_−
6	3.92	2H, −CH_2_−
7	5.25	H, −CH=
8	5.88	2H, =CH_2_

**Table 3:** Analytical table of the ^1^H-NMR spectrum for bis[(3-ethyl-3-methoxyoxetane)propyl]diphenylsilane.

Location of H	Chemical Shift/(δ/ppm)	Structural Formula
1	7.36	2H, –CH–
2	7.36	4H, –CH–
3	7.54	4H, –CH–
4	1.54	4H, –CH_2_–
5	1.73	4H, –CH_2_–
6	3.37	4H, –CH_2_–
7	3.29	4H, –CH_2_−
8	4.65	4H, –CH_2_–
9	4.65	4H, –CH_2_–
10	1.25	4H, –CH_2_–
11	0.96	6H, –CH_3_–

**Table 4:** Data of Photo-DSC.

	Bis[(3−ethyl−3−methoxyoxetane)propyl]diphenylsilane	E-51	2021 P
Induction time (s)	0.20	9.00	0.15
t_max_ (s)	12.00	113.40	11.70
Total heat (J)	53.42	101.03	82.59
H_max_ (J/g)	26.40	65.90	43.50

**Table 5:** Data of testing tensile property for the cured film.

	Tensile Strength/MPa	Elastic Modulus/MPa	Elongation at Break/%
1	72.3	19,002.3	4.7
2	78.8	19,583.2	4.8
3	74.5	19,239.6	4.9
4	74.2	19,709.6	4.5
5	77.7	19,673.1	4.6
Average value	75.5	19,441.6	4.7
Standard deviation	2.6	307.9	0.2

**Table 6:** Data of bending mechanical property for the cured film.

	Bending Strength/MPa	Elastic Modulus/MPa	Elongation at Break/%
1	49.8	1845.4	6.5
2	52.6	1854.8	6.7
3	47.8	1847.6	6.4
4	48.3	1858.6	6.4
5	48.8	1864.0	6.6
Average value	49.5	1854.1	6.5
Standard deviation	1.9	7.7	0.1

**Table 7:** Light transmittance of bis[(3-ethyl-3-methoxyoxetane)propyl]diphenylsilane.

Wavelength/nm	350	400	450	500	550	600	650	700
Transmittance/%	98.42	99.01	99.29	99.20	99.28	99.40	99.27	99.01

## Data Availability

All data is offered by corresponding author for reasonable request.
